# Semiconductor Quantum Dots in Chemical Sensors and Biosensors

**DOI:** 10.3390/s90907266

**Published:** 2009-09-10

**Authors:** Manuela F. Frasco, Nikos Chaniotakis

**Affiliations:** Laboratory of Analytical Chemistry, Department of Chemistry, University of Crete, Vassilika Voutes, 71003 Iraklion, Crete, Greece; E-Mail: mffrasco@chemistry.uoc.gr

**Keywords:** semiconductor quantum dots, nanoassembly, optical sensors, photoluminescence

## Abstract

Quantum dots are nanometre-scale semiconductor crystals with unique optical properties that are advantageous for the development of novel chemical sensors and biosensors. The surface chemistry of luminescent quantum dots has encouraged the development of multiple probes based on linked recognition molecules such as peptides, nucleic acids or small-molecule ligands. This review overviews the design of sensitive and selective nanoprobes, ranging from the type of target molecules to the optical transduction scheme. Representative examples of quantum dot-based optical sensors from this fast-moving field have been selected and are discussed towards the most promising directions for future research.

## 1. Introduction

**Figure 1.**
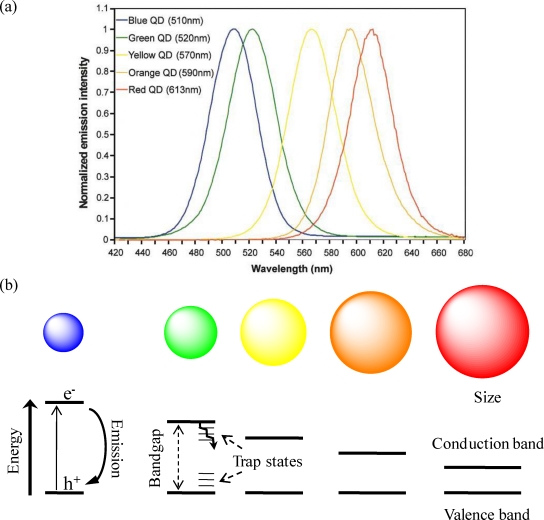
(a) Emission spectra of CdSe/ZnS QDs (water-soluble QDs excited at 350 nm) (Reprinted with permission from Macmillan Publishers Ltd., *Nature Biotechnology* [7], Copyright 2002); (b) Illustration of size-tunable QDs and creation of the exciton (electron-hole pair) upon photoexcitation followed by radiative recombination (fluorescence emission) or relaxation through trap states.

Quantum dots (QDs) are colloidal nanocrystalline semiconductors possessing unique properties due to quantum confinement effects. QDs typically have very broad continuous absorption spectra, at wavelengths extending from the ultraviolet to the visible, depending on the particle size. The broad excitation and narrow size-tunable emission spectra (usually 20–40 nm full width at half maximum intensity), negligible photobleaching, and high photochemical stability are some of their remarkable properties [1,2] (Figure 1). Fluorescence is a powerful tool in biological research, which relevance relies greatly on the availability of sensitive and selective fluorescent probes. The routinely employed chemical fluorophores usually have some drawbacks, including susceptibility to chemical changes in the medium and to photobleaching, fixed emission spectra, and limited Stokes shift (separation between excitation and emission maxima). The optical and spectroscopic unprecedented features of QDs make them very convincing alternatives to traditional fluorophores in a range of applications, including for multiplex bioanalysis. An extensive comparison of the advantages and limitations of both classes of fluorescent labels has been recently reviewed [3].

QDs have bandgap energies that vary as a function of size (the higher size of the QD, the lower the energy of the bandgap). The bandgap energy corresponds to the minimum energy that must be provided to move an electron from the valence to the conduction band creating a hole behind. The created electron–hole pair, an exciton, may recombine immediately to produce heat or light with energy equal to the bandgap energy, but it is more likely that trap states within the material trap either the electron or the hole [4] (Figure 1). Therefore, the critical influence of QDs surface on photoluminescence can be understood in terms of the trap states, which can be caused by structural defects, such as atomic vacancies, local lattice mismatches, dangling bonds, or adsorbates at the surface [5]. The excited electron or hole can be trapped by these local energy minima and lead to nonradiative recombination. In order to decrease the possibility of charge carriers to be kept in trap states, surface passivation has been achieved for example by overcoating the QDs with a wider bandgap semiconductor quantum shell (e.g., CdSe coated with ZnS as a core-shell nanocomposite) [6]. This increases the photostability of the core and the quantum yield, so that core-shell structured QDs are more favourable for fluorescence-based applications [7].

Due to the availability of precursors and the simplicity of crystallization, Cd-chalcogenide nanocrystals have been the most studied colloidal QDs. Several synthetic methods have been reported since the first synthesis of monodisperse CdE (E = S, Se, Te) nanocrystals [8] and extensive reviews on the synthesis of colloidal nanocrystals can be found [9,10]. Usually, QDs are synthesized using a coordinating solvent (e.g., trioctylphosphine oxide/trioctylphosphine, TOPO/TOP) that makes them soluble in organic solvents and serves as stabilizer by preventing their agglomeration. For the majority of the biological applications, these hydrophobic nanocrystals need to be transferred to aqueous solutions using various routine methods, such as ligand exchange, surface silanization, embedding in a polymer shell or incorporation in micelles [11]. Therefore, the knowledge of the surface chemistry of QDs is needed to understand their optical properties and to manipulate them to achieve a desired application.

Owing to the above mentioned fascinating optoelectronic properties, important applications of these nanomaterials are vast and some of them will not be covered in this review. Together with the growing ability to modify the surface of QDs by conjugation with appropriate functional molecules and with the progressive knowledge on their toxicity and long-term fate on live organisms, *in vivo* labelling, imaging and therapeutic applications in biomedicine are in expansion [12,13]. There are ongoing efforts to extend their *in vivo* suitability by developing QDs of more biocompatible materials and able to emit in the near-infrared region of the spectrum to take advantage of the improved tissue penetration depth and reduced background fluorescence at these wavelengths. QDs have already been tagged to multiple different biomolecules, proving their potential to provide information on disease-related molecular events essential for diagnosis and treatment [14,15]. Thus, QD technology holds a great potential for *in vivo* bioanalysis and recent overviews on this subject can be found [16,17]. Chemical and biosensing have also been taken advantage of the new functional platform provided by QDs, as demonstrated by numerous works summarized in the literature [18,19]. Herein, the emphasis will lie on the progress in the use of nanoassemblies incorporating QDs as fluorescent probes in chemical sensors and biosensors. First, the optical transduction schemes most commonly used are discussed. Subsequently, the design of a broad range of tailored QD-bioconjugates enabling sensitive, selective and multiplex sensing is highlighted by giving an overview of QD-based optical probes according to the type of target analytes.

## 2. Optical Transduction

As previous referred the novel optical properties of QDs make them ideal nanomaterials for ultrasensitive and multiplexing applications in optical sensing. As the luminescence of QDs is very sensitive to their surface states, fluorescence transduction is based on the principle that chemical or physical interactions occurring at the surface of the QDs change the efficiency of the radiative recombination, either leading to photoluminescence activation or quenching [5]. Following this approach, the changes induced by the direct interaction between the analyte and the QDs surface, unmodified or functionalized with a given ligand have supported the selective detection of a multitude of compounds.

Many of the designed sensing assemblies do not make use of QDs merely as passive labels. Instead, these systems are based on energy flow such as transfer of electronic excitation energy between the components of such nanoassemblies. This can occur when light energy absorbed by QDs (donor) is transferred to a nearby acceptor species, such as an organic fluorophore (acceptor) in a process called Förster (Fluorescence) Resonance Energy Transfer (FRET) [20]. The rate of energy transfer depends on the distance between the donor and the acceptor, their relative orientations, and the spectral overlap. Therefore, the energy flow at the nanoscale can be altered, set up or disrupted, by small perturbations such as specific interactions due to molecular binding or cleavage events [21]. Since the first study demonstrating the energy transfer from QDs to organic chromophores [22], many works have been developed using QDs as a scaffold for FRET assays. According to Clapp and collaborators [23], a good FRET efficiency from an organic flurophore (donor) to QDs (acceptor) is not expected because QDs are poor acceptors of energy. Upon exciting the donor there is a coincident excitation of QDs due to their broad excitation spectrum. Thus, the reduced energy transfer rate prevents the detection of QDs FRET enhancement. The relatively slow QD decay rate also diminishes QDs ability as acceptor. However, some examples of QDs functioning as good energy acceptors emerge [24], and further research on the circumstances under which QDs might function as efficient acceptors of energy is necessary.

Bioluminescence Resonance Energy Transfer (BRET) is a principle ideally suited for luminescent QDs because it eliminates the difficulties encountered in using QDs as acceptor fluorophores. BRET exploits the photon generating process from a chemical reaction to transfer the excitation energy nonradiatively to a proximal fluorescent acceptor. When a light-emitting protein (donor) nonradiatively transfers energy to QDs (acceptor), self-illuminating QD-conjugates are created with no requirement for external excitation light for QDs to fluoresce [25]. Analogously to FRET, this nonradiative transfer of energy requires the donor/acceptor to be in close proximity.

Although fluorescence and FRET have been the most explored transduction schemes so far, other optical methods such as BRET, Photoinduced Electron Transfer (PET), or phosphorescence are in expansion. Comprehensive examples on particular applications of each method will be presented.

## 3. QD-Based pH Probes

The identification of pathways to obtain hybrid nanoconjugates by capping inorganic QDs with organic ligands lead to the design of pH-sensitive QDs with very promising applications for a variety of analytical purposes, particularly for the development of luminescent chemosensors. Within this strategy, the surface of CdSe/ZnS QDs was capped with an organic compound incorporating a dithiolane anchoring group, an electron-rich indole, a 4-nitrophenylazophenoxy chromophore and a 1,3-oxazine ring in their skeleton [26]. The pH sensing is based on the 1,3-oxazine ring that opens upon addition of base or acid. In the first case, the chemical stimulation generates a 4-nitrophenyl-azophenolate chromophore and is transduced into luminescence quenching due to PET from the QDs to the chromophores adsorbed on their surface. When the 1,3-oxazine ring opens in acidic pH it results in 4-nitrophenylazophenol that does not absorb in the visible region, i.e., the chromophore does not accept the excitation energy from the QDs and is also a poor electron donor. As a result, there is an increase in the quantum yield. The modulation of this reversible switch of the luminescence is thus very promising for the design of sensing devices towards other analytes.

A different approach was based on FRET between CdSe/ZnS QDs encapsulated within an amphiphilic polymer and a pH-sensitive squaraine dye conjugated on the cap surface. As the absorption spectrum of the dye is pH dependent, so is the FRET efficiency and the ratio of QDs to dye emission becomes a function of pH [27]. There are also some works based on thioalkyl-functionalized QDs, which are pH-sensitive [28], suggesting many different biological applications. In this context, mercaptoacetic acid (MAA)-CdSe/ZnSe/ZnS QDs have been used as an intracellular pH sensor by observing a quenching of QDs fluorescence in acidic pH [29]. This strategy of using core/shell/shell structure makes the QDs more robust against photobleaching, while in combination with surface modification enables a long-term monitoring of intracellular pH. The use of pH-sensitive mercaptopropionic acid (MPA)-capped QDs was successful in following enzymatic reactions, by monitoring the hydrolysis of glycidyl butyrate catalyzed by porcine pancreatic lipase [30], and in the quantification of drugs, such as acidic tiopronin [31]. A simple method has been proposed for the detection of urea based on the pH-sensing abilities of mercaptosuccinic acid-CdSe/ZnS QDs in a system also containing the enzyme urease [32]. The urease-catalyzed hydrolysis of urea generates hydroxide anions that gradually increase the pH value of the solution, leading to an enhancement of QDs fluorescence that could be correlated with increasing concentrations of urea. These studies are revealing of the long path for future applications of QDs as pH probes.

## 4. Ion Sensors

Sensing of ions via analyte-induced changes in photoluminescence of QDs is a very active research field and concurrent thiol-based capping strategies have emerged. These include the use of a number of thioalkyl acid ligands (e.g., MAA, also named thioglycolic acid, TGA; dihydrolipoic acid, DHLA). As the self-assembly proceeds via metal-thiol affinity interaction, the carboxyl groups get exposed to the surrounding aqueous solution. The ligand is thus simultaneously responsible for participating in the response to metal ions and for the solubility of QDs in the aqueous environment. The usefulness of QDs as ion probes has also been assayed using different capping agents such as l-carnitine or bovine serum albumin [33,34]. Luminescent QDs as probes for ions in physiological buffer samples was first demonstrated by Chen and Rosenzweig [35] that achieved selectivity by changing the nature of the capping layer of CdS QDs. The optical properties of QDs functionalized with l-cysteine proved to be dependent on Zn^2+^ with an increase of luminescence, while thioglycerol-CdS QDs were quenched in the presence of Cu^2+^ [35]. In general, the interaction of ions with QDs induces a fluorescence quenching that can be attributed to inner-filter effects, nonradiative recombination pathways and electron transfer processes [36]. However, fluorescence enhancement can also be observed and passivation of trap states or defects on the surface of the QDs is usually the mechanism ascribed for the observed increase in quantum yield [37]. Surface ligands play a critical role in ion selectivity and have significant effects on fluorescence response of QDs to metal ions. This was evidenced in a systematic study with three different thiol-capped CdTe QDs, using MAA, l-cysteine and reduced glutathione as ligands. The effects of cations on fluorescence intensities were similar only to some extent depending also on the cation [38].

A sensor for Hg^2+^ has been developed based on a selective fluorescence quenching of CdSe/ZnS QDs modified with sulphur calixarene [39]. This system showed a detection limit in the nM range and the influence of other metal ions was very weak with the exception of Pb^2+^ at higher concentrations that produced a measurable quenching of QDs fluorescence. Another example is the FRET-based system developed between TGA-CdTe QDs and the acceptor butyl-rhodamine B in the presence of a surfactant (cetyltrimethylammonium bromide) to increase FRET efficiency [40]. Upon addition of Hg^2+^ the fluorescence of both donor and acceptor was quenched. Since the fluorescence of free butyl-rhodamine B was not affected by the cation, the quenching was attributed to the binding of Hg^2+^ onto the surface of the TGA-CdTe QDs and the detection relied on the spectral change of the acceptor. Similar to the previous described method, the detection limit was in the nM range, and the method proved to be reproducible even in complex matrices where possible interferents had negligible effects on the determination of Hg^2+^.

Recently, Zn^2+^ selective nanosensors based on PET fluorophore-ligand have been designed using azamacrocycles, a class of nitrogen-containing analogues to crown ethers, conjugated via an amide link to MPA-CdSe/ZnS QDs [41]. The interaction of the photoinduced QDs charge carriers with the azamacrocycles disrupted the radiative recombination process, most likely because the lone pair electrons of the nitrogen atom stabilized the transfer of the hole from the QDs to the azamacrocycle. The restoration of the fluorescence intensity occurred when zinc ion entered the azacrown, requiring the involvement of the nitrogen atom in the coordination, which became unavailable for participating in the charge separation process [41]. These ion-sensitive QDs showed good selectivity to zinc in comparison to other cations, and their applicability for the determination of zinc in physiological media was demonstrated.

Comparatively, the use of luminescent QDs in the selective detection of anions has been almost unexplored. For example, CdSe QDs have been functionalized with 2-mercaptoethane sulfonate for optical detection of cyanide ions due to quenching of the fluorescence, reaching a limit of detection in the μM range [42]. More recently, a PET-based platform has been proposed for anion detection using CdSe/ZnS QDs capped with a charge neutral thiourea receptor [43]. In a format typical of PET, QD-thiol terminated ethyl spacer-thiourea conjugate enabled the detection of tetrabutylammonium salts of fluoride, chloride and acetate in chloroform. There was an increase in PET from the excited QDs to the receptor and thus a quenching of fluorescence due to an increase in the reduction potential of the receptor upon anion binding.

Some works have also demonstrated the applicability of QDs surface modification for increasing the selective sensing of multiple ions simultaneously. The pentapeptide (Gly–His–Leu–Leu–Cys) coated CdS QDs showed to respond with high selectivity to Ag^+^ and Cu^2+^, which induced fluorescence quenching in aqueous media [44]. A recent work designed CdSe/ZnS QDs conjugates with an organic receptor, a Schiff base, allowing chromogenic selective detection of Cu^2+^ and Fe^3+^ simultaneously in semi-aqueous solution [45]. Triethanolamine (TEA)-capped CdSe QDs showed to be responsive through an effective fluorescence quenching only in the concomitant presence of Hg^2+^ and I^−^ [46]. It was hypothesized that I^−^ bridged the binding between QDs and Hg^2+^, allowing the formation of a stable complex and an effective electron transfer from the QDs to the cation and consequent fluorescence quenching.

## 5. Detection of Organic Compounds

**Figure 2.**
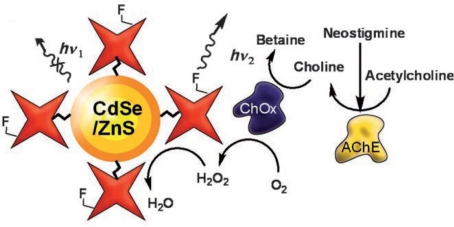
QD-based sensor for anti-acetylcholinesterase (AChE) pesticides: fluorescence of fluorophore-modified avidin-capped CdSe/ZnS QDs is quenched by hydrogen peroxide (H_2_O_2_) generated by the AChE-choline oxidase (ChOx) biocatalytic cascade; in the presence of a pesticide such as the carbamate neostigmine, AChE activity is inhibited, less H_2_O_2_ is produced and QDs fluorescence quenching decreases (Reprinted with permission from [49], Copyright 2008 Wiley-VCH Verlag GmbH & Co. KGaA).

Pesticides are still commonly used in agricultural practices, despite the health and environmental problems that can result from accumulated levels of these compounds. QDs and immunological techniques were combined to detect and quantify the herbicide 2,4-dichlorophenoxyacetic acid (2,4-D) [47]. The enzyme alkaline phosphatase (ALP) was conjugated to the herbicide 2,4-D, followed by the formation of an amide bond between the carboxylic group of MPA-CdTe QDs and the amine group of ALP. The quantitative analysis of 2,4-D was then performed based on a fluoroimmunoassay biosensor [47]. MPA-CdSe/ZnS electrostatically conjugated to an organophosphorous hydrolase was used to detect paraoxon, which quenched the photoluminescence of the QDs probably due to a change in the secondary structure of the enzyme upon pesticide binding [48]. QDs sensitivity to hydrogen peroxide (H_2_O_2_) elicited the development of versatile fluorescent QD-based sensors potentially applicable to a number of oxidases that biocatalyze the generation of H_2_O_2_ [49]. The inhibition of acetylcholinesterase (AChE) by the carbamate neostigmine exemplified the proposed system. In this hybrid method AChE was responsible for hydrolysing acetylcholine to choline and choline oxidase subsequently oxidised choline to betaine with the concomitant generation of H_2_O_2_ and quenching of the fluorescence of CdSe/ZnS QDs (Figure 2). In the presence of the inhibitor, the biocatalytic cascade was interrupted and QDs fluorescence was not quenched.

Expanding knowledge of the surface chemistry of this nanomaterial allowed the development of QD-based sensors to detect analytes resulting from industrial or warfare applications. QDs fluorescence was modulated in the presence of nitroaromatic compounds or diamines, providing new pathways to assess these and related compounds [50,51]. The conjugation of QDs to histidine tail engineered proteins has been applied in self-assembly of a recombinant specific antibody of the explosive 2,4,6-trinitrotoluene (TNT) onto QDs surface allowing the detection of this compound by competitive immunoassay [52]. The fluorescence quenching of QDs by various detergents of common use showed the role of the surface charge in the quenching process and has been explained on the basis of nonradiative recombination with deep-traps, less mobile holes and by stabilizing electrons in surface traps [53,54]. The applicability of TGA-CdTe QDs for the determination of cationic surfactants in real samples was satisfactory, showing good sensitivity and selectivity [54]. Functionalized QDs with cyclodextrins, cyclic oligosaccharides with a variable-sized hydrophobic internal cavity for the formation of inclusion complexes and a hydrophilic external surface to retain the aqueous solubility of the macrocycle, have been proposed as selective probes for fluorescent determination of various polycyclic aromatic hydrocarbons [55].

## 6. Detection of Biomolecules

### 6.1. Nucleic Acids

**Figure 3.**
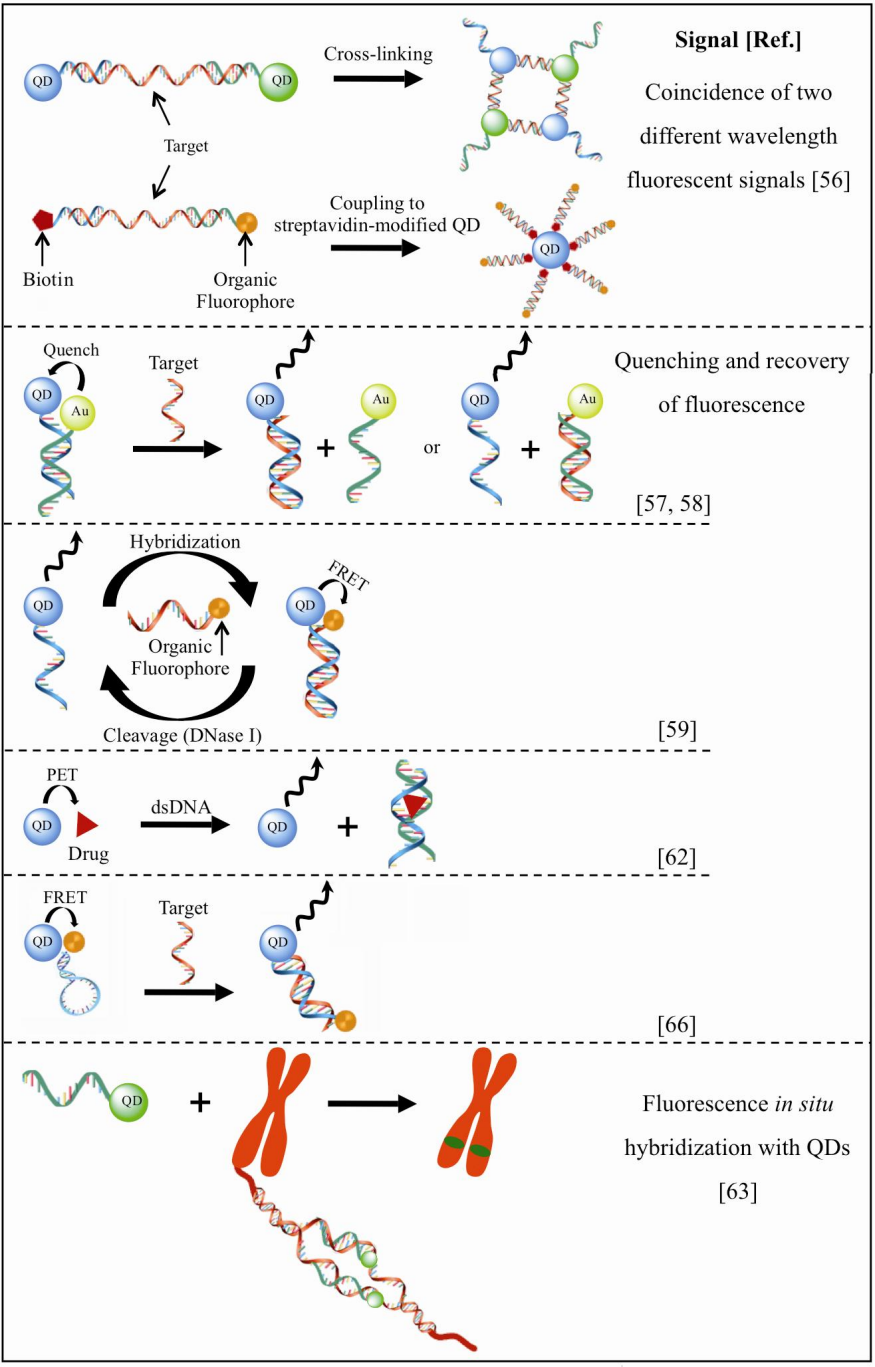
Illustration of various QD-based sensing configurations for nucleic acids.

Various DNA sensing approaches with good selectivity and reduced levels of interfering molecules have been developed. For instance, two concepts were designed based on the specificity of hybridization between target-probe oligonucleotides [56]. In both methods, the detection of targets required the coincidence of two different wavelength fluorescent signals emitted from the QD/DNA target-probe complexes (Figure 3). The first used two QDs with distinct emission wavelengths, modified with two single-stranded (ss) DNA probes via a biotin-streptavidin interaction. These DNA probes were designed to hybridize at different binding sites of the same target DNA strand, so that upon target-probe hybridization the cross-linking of the QDs occurred. In the second approach, two ssDNA probes, one biotinylated and the other conjugated to an organic fluorophore, were first mixed with the DNA target to form a sandwich hybridization structure that was captured by the streptavidin-modified QD (only one type of QD was used). As the organic fluorophore-labelled duplex structures were coupled to a QD, besides functioning as fluorescent tags QDs also worked as nanoscaffolds resulting in amplified signals [56]. In another scheme based on the specificity of oligonucleotide hybridization, ssDNA was end-linked to QDs, while a complementary chain was conjugated to gold nanoparticles (AuNPs). The fluorescence quenching of QDs tagged with the oligonucleotide chain occurred by close contact with the AuNP as the two chains hybridized. In the presence of the target DNA sequence with higher complementarity, the double-stranded (ds) DNA of the probe opened and the fluorescence of QDs could be recovered [57,58] (Figure 3).

DNA hybridization and cleavage processes have also been studied [59]. The hybridization was monitored by following FRET between QDs and a molecular fluorophore, whereas treatment of the QD/dye-DNA structure with deoxyribonuclease (DNase I) cleaved the DNA duplex and restored the fluorescence properties (Figure 3). The dynamics of DNA replication and telomerisation have also been reported [60]. MPA-CdSe/ZnS QDs were modified with the thiolated oligonucleotide and later incubated with a mixture of deoxyribonucleotides with an organic fluorescent dye in the presence of telomerase. The fluorescence emission of the QDs decreased as telomerisation proceeded, with the concomitant increase of the characteristic emission of the fluorophore by FRET from the QDs to the dye molecules incorporated into the telomeric units by telomerase [60]. Using a similar procedure but in the presence of a polymerase, the replication process could be followed by FRET from the QDs to the incorporated dye unit and allowed the detection of a viral DNA [60].

Unlike these reports, there are also FRET-based DNA hybridization sensors that do not require covalent immobilization of the probe molecules, minimizing in this way DNA modification. Water-soluble CdTe QDs were prepared using TGA as the capping ligand, and this negatively charged QDs were further incorporated in a cationic polymer solution of poly(diallyldimethylammonium) as the electrostatic linker. The resultant positively charged QDs acted as FRET donors to dye acceptor-labelled ssDNA. The hybridization recognition was based on the interaction of ssDNA and dsDNA with the functionalized QDs leading to differential changes of FRET efficiency [61].

From a biomedical point of view, exploiting QD technology to improve the detection of the interaction between DNA and anticancer drugs is of high interest. A recent study, making use of a model anthraquinone anticancer drug (mitoxantrone, MTX), demonstrated the usefulness of a PET-based sensor to study the interactions between MTX and DNA. The MTX adsorbed on QDs quenched their photoluminescence through an electron-transfer process from QDs to MTX, which could then be restored in the presence of DNA that bound MTX and removed it from the surface of QDs [62] (Figure 3). With this sensing model there was no need for labelling, modifying or immobilizing processes and a good sensitivity of detection was achieved. QD-oligonucleotide-based probes have also been used to detect chromosome abnormalities or mutations using an adaptation of FISH (fluorescence *in situ* hybridization) procedures by incorporation of QDs [63]. CdSe/ZnS QDs functionalized with hydroxyl groups were coupled with oligonucleotide sequences of interest via a carbamate linkage, leading to photostable QD-probe conjugates with strong emission and useful for FISH assays (Figure 3). QD-DNA conjugates have also been used in a microarray format as efficient probes of single-nucleotide polymorphism and for multiallele detection [64].

A FRET mechanism between QDs and organic dyes has been successfully employed in RNA-peptide interaction studies. The specific interaction between biotinylated RNA and the cyanine dye-labelled peptide of interest originated a complex that was then assembled with streptavidin-QDs in a way that QDs functioned both as nanoscaffold and FRET donor. A broader application of this alternative method is particularly interesting concerning the relevance of gene expression regulation and development of new drugs [65]. The amenable application of reactive peptides was also explored in the self-assembly of DNA to QDs. The hybrid QD-DNA-dye molecular beacon was attained using a thiol-reactive hexahistidine peptidic linker that was chemically attached to thiolated-DNA oligomers. An efficient FRET between the QD-donor and the dye-acceptor was based on a labelled hairpin DNA stem structure bringing both in close proximity. This distance was altered and FRET efficiency disrupted in the presence of complementary DNA by unwinding the stem-loop structure so that specific and nonspecific target DNA could be discriminated [66] (Figure 3).

### 6.2. Proteins and Enzymes

The first report on the conjugation of protein molecules to luminescent CdSe/ZnS QDs arose from Mattoussi’s group [67]. In this study, DHLA capped-QDs, i.e., negatively charged QDs were electrostatically bound to a designed chimeric fusion protein based on *Escherichia coli* maltose binding protein (MBP)-basic leucine zipper. This electrostatic noncovalent approach has been further extended towards the preparation of bioconjugates of CdSe/ZnS QDs with antibodies to be applied in fluoroimmunoassays: (a) using a genetic fusion between the immunoglobulin G (IgG)-binding *β*2 domain of streptococcal protein G (PG) with the positively charged leucine zipper, which allows the readily formation of QD-IgG bioconjugates; (b) using the positively charged avidin to form QD-antibody conjugates by reaction with biotinylated IgG [68]. For example, these conjugates were applied in fluoroimmunoassays for the detection of protein toxins and, making use of four QDs with various emission peaks coupled with antitoxin antibodies, multiple toxins could be detected simultaneously [69]. Another approach was based on the direct conjugation of CdSe/ZnS QD-IgG complexes using a genetically engineered tripartite fusion protein. This fusion protein was comprised of a histidine tag for QD conjugation, an elastin-like peptide for stimuli-responsive purification and the protein L (cell-wall component of *Peptostreptococcus magnus*) that has high affinity to IgG [70]. The functionality of this sensitive immunofluorescent probe was demonstrated in the detection of a representative tumour antigen.

Recently, CdSe/ZnS QDs conjugated to ssDNA-fluorescent dye labelled, enabled the development of a QD-FRET probe for the quantitative determination of micrococcal nuclease (MNase) [71]. It has been reported that MNase is the extracellular nuclease of *Staphylococcus aureus*. Therefore, evaluating the content in MNase may be used to evaluate the pathogenicity of this bacterium. The QDs where conjugated to ssDNA through streptavidin-biotin affinity ligation. The FRET occurring between the QDs and the fluorescent dye present at the other end of the ssDNA was disrupted in the presence of MNase, which digested the ssDNA. The restoration of QDs fluorescence enabled the quantification of MNase in solution and served as starting point for further determinations in culture medium of *S. aureus*.

Many of the possible applications of peptide capped-QDs include monitoring alterations of enzymatic activities, which can be related to important biological processes and to diseases. An excellent example was the design of QD bioconjugates able to detect proteolytic activity. By attaching dye- or AuNP-labelled peptide structures containing appropriate cleavage sequences to QDs, proteases activities and their modulation (e.g., collagenase, thrombin, chymotrypsin) were detected upon specific cleavage of the peptide and alteration of FRET signal [72,73]. Recently, a QD-fluorescent protein conjugate was developed to detect the presence of caspases, essential for apoptosis. The fluorescent protein mCherry was modified to express a target peptide sequence with a caspase 3 cleavage site and a histidine tag to be self-assembled on DHLA-modified QDs [74]. In the presence of caspase enzymes, the linker was cleaved and thus the FRET efficiency between the QD and the mCherry acceptor was reduced.

The capping chemistry with DNA aptamers can also serve the purpose of providing selective binding to a protein target. For example, a DNA aptamer was shown to passivate PbS QDs, rendering them water-soluble and stable against aggregation, while retaining the secondary structure needed to selectively bind to its thrombin target [75]. The selective binding of the target to the aptamer-functionalized QDs, resulted in a quenching of the photoluminescence that was attributed to charge transfer from functional groups on the protein to the QDs. In another study, thrombin-binding DNA aptamer was coupled to QDs and a quencher labelled antisense oligonucleotide was used to maintain the aptamer in an inactive form by disrupting its structure. The addition of thrombin released the antisense DNA strands and the fluorescent signal was generated [76].

Another interesting design to analyse protein-ligand interactions involved the use of a quencher functioning as PET acceptor electrostatically associated to the surface of the QDs [77]. The QDs luminescence could be restored in the presence of an appropriate receptor able to bind and remove the quencher from the QDs surface. The viability of this approach was demonstrated by using MPA-CdSe/ZnS QDs and a compound integrating a bipyridinium-based quencher and a biotin ligand in the same molecular skeleton. The luminescence enhancement was then observed specifically upon addition of streptavidin.

**Figure 4.**
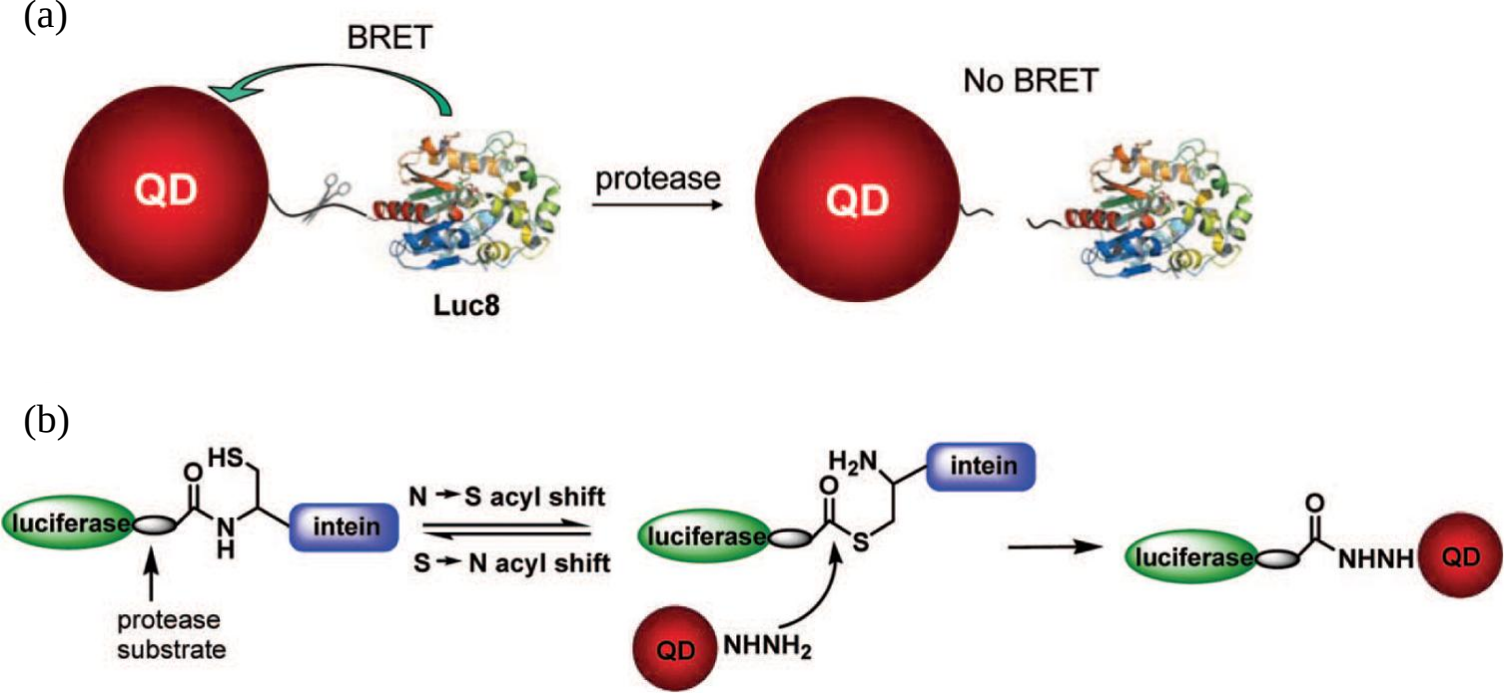
Schematic representation of QD BRET-based sensors for proteolytic activity of matrix metalloproteinases (MMPs): (a) QDs are linked to a bioluminescent mutant of *Renilla* luciferase protein (Luc8) through an MMP peptide substrate; (b) Intein, a polypeptide sequence able to excise itself and rejoin the remaining portions with a peptide bond, mediates site-specific conjugation of Luc8 fusion proteins to QDs through catalyzing the splicing reaction via formation of an active thioester intermediate (Reprinted with permission from [79], Copyright 2008 American Chemical Society).

Conjugation of luminescent proteins to QDs has also been the focus of research. This type of approach is based on BRET where QDs serve as energy acceptors for the light emitting protein (e.g., bioluminescent protein *Renilla* luciferase) [78]. The QD conjugates can emit light without light excitation, since the energy released in the oxidation of luciferase substrate is transferred to the QDs via BRET, generating light emission from the QDs. This method has been proven useful for detecting the activity of matrix metalloproteinases (MMPs) [79]. In the presence of the target MMP, the substrate linker responsible for bringing luciferase and QDs into close proximity was cleaved and BRET was disrupted (Figure 4).

Multiplex fluoroimmunoassays have also been developed based on electrostatic adsorption of antibodies on the outermost surface of CdTe QD-encoded polystyrene microspheres. Two kinds of functional multicolour microspheres were prepared with two different antibodies, anti-human IgG and anti-rabbit IgG. The respective target antigens could then be detected synchronously with one excitation wavelength because the microspheres could be distinguished from each other based on their fluorescence signals [80].

There are strategies in progress for developing multiplex FRET-based systems for distinct targets. The activity of protease was monitored through FRET annihilation on cleavage of a chimeric green fluorescent protein (GFP) with an inserted sequence recognized by a protease (e.g., trypsin) to release GFP from QDs surface. The same principle was applied to follow DNase activity upon digestion of fluorophore-modified dsDNA bound to QDs. A third example was presented for DNA polymerase, with FRET occurring as fluorescent-labelled nucleotides become incorporated to extend the ssDNA attached to the QD [81]. Two of these systems, with both cleavage enzymes, proved to operate simultaneously and independently in the same working solution upon excitation at a single wavelength, supporting further developments on the application of other multiplexing systems.

### 6.3. Other Molecules of Biological Interest

There is great interest in developing nanosensors to detect amino acids, known for their relevant roles in metabolism and as building blocks of proteins. In a straightforward study, MAA-QDs proved to be sensitive and selective fluorescent probes of l-cysteine (limit of detection in the nM range) [82]. Proving their versatility for surface coating of QDs, *p*-sulfonatocalix(*n*)arene (*n* = 4 and 6) allowed the detection of methionine and phenylalanine, respectively, in physiological buffer solution through enhancement of the capped CdSe QDs fluorescence [83]. Other strategies to sense amino acids include the development of cyclodextrin-capped QDs [84]. The chiral recognition of two amino acids, tyrosine and methionine, was possible by the high enantioselective response of the cyclodextrin-CdSe/ZnS QDs. Within a certain concentration range, one of the enantiomer of the chiral amino acid enhanced the fluorescence intensity, whereas the other had a negligible effect. This differentiation between the naturally occurring l forms and the d forms that may indicate negative symptoms or disease is a first step in the development of QD-based chiral sensors useful to study metabolic and regulatory processes.

The fluorescence quenching of QDs has been used to detect a number of other compounds of biological interest, such as the diuretic drug spironolactone [85]. In one of the few reports on phosphorescence changes of QDs, the detection of enoxacin, a quinolone antibiotic, relied on the quenching of the phosphorescence emission of Mn-doped ZnS QDs [86]. Multiplex analysis has always been a challenge and a sensor based on encoded aptamer-linked nanostructures (with QDs and AuNPs) has been designed and assayed against adenosine and cocaine [87]. The simultaneous quantification of these molecules relied on the use of two QDs with emission peaks at different wavelengths. The QDs fluorescence was quenched when assembled to AuNPs by the selected aptamers against each target molecule. Upon addition of the target analyte the nanostructures were disassembled and the emission of QDs restored. Adenosine triphosphate (ATP) has been detected using CdSe/ZnS QDs thiol-functionalized with a charged quaternary ammonium group. This group ensures water solubility and is responsible for discriminating between nucleotides based on differences in their surface charge and thus on their attraction to the surface of the QDs [88]. The fluorescence modulation was achieved when the interaction between the negatively charged ATP and the positively charged QD lead to an electron transfer and a quenching of the fluorescence. This example of fluorescent probe showed good selectivity towards ATP among other nucleotides and is a promising tool for ongoing improvements. Another optical system has been developed for the detection of the neurotransmitter acetylcholine using surface-coated CdSe/ZnS QDs with amphiphilic *p*-sulfonatocalix[4]arene that hosted acetylcholine molecules resulting in the selective quenching of QDs fluorescence [89].

**Figure 5.**
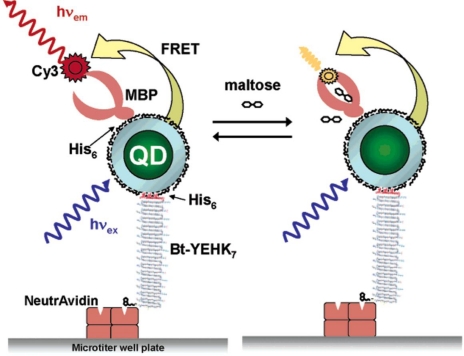
Schematic illustration of a FRET-based surface-immobilized maltose biosensor: QDs captured in NeutrAvidin-coated microtiter well plates functionalized with biotinylated peptide (Bt-YEHK_7_) were subsequently self-assembled with a mutant dye-labelled maltose binding protein (MBP), enabling the detection of maltose due to changes in Cy3 emission (Reprinted with permission from [92], Copyright 2006 American Chemical Society).

QD-based optical sensors have been designed to monitor carbohydrates like maltose or glucose. Engineered MBP with an oligohistidine tail, labelled with an acceptor dye at the allosteric site (e.g., cyanine Cy3), was noncovalently self-assembled on DHLA-capped QDs and applied in FRET-based assays for monitoring maltose [90]. The assembly was further immobilized on NeutrAvidin-coated glass substrates using biotinylated MBP or biotinylated IgG that acted as bridge between NeutrAvidin and avidin-MBP-QD [91]. In another immobilization strategy, a peptide linkage was designed to be biotinylated at one terminus allowing anchorage to the NeutrAvidin substrate, while an oligohistidine sequence was appended at the other terminus to bind at the surface of DHLA-QDs [92] (Figure 5).

One of the many QD-based strategies developed to detect glucose was based on the covalent conjugation of glucose oxidase (GOx) to CdTe QDs. The mechanism for glucose sensing relied on the fluorescence quenching of CdTe QDs, which was caused by H_2_O_2_ produced during GOx-catalyzed oxidation of glucose. An electron-transfer reaction occurred at the surface of the CdTe QDs where H_2_O_2_ was reduced to O_2_, which in turn lied in electron/hole traps on CdTe QDs and could be used as a good acceptor, thus quenching CdTe QDs fluorescence. The produced O_2_ participated then in the catalyzed reaction of GOx, forming a cyclic electron-transfer mechanism of glucose oxidation, which was favourable for the whole reaction system [93].

## 7. Conclusions and Outlook

QDs have extraordinary electronic and optical properties that have been investigated for over a decade, making them one of the most prolific nanomaterials. These semiconductor nanocrystals have a broad variety of applications that include their use as a new type of fluorescent probes as well as active components in nanostructure-biomolecule complexes. Various optical transduction schemes have already been successfully explored for sensing a wide range of molecules, allowing low levels of detection and reduced interference of other compounds in complex samples. Currently a reality, multiplex detection is an ever-expanding research tool for QDs in optical arrays. Nonetheless, the vast majority of applications occur in bulk solutions limiting the development of reusable sensors, and routine analytical applications are hindered so far due to their known high toxicity. These will certainly be two of the challenges tackled in the near future concomitant with the continuous progress in the synthesis and conjugation methods for increased stability, sensitivity and binding specificity of QD-nanoassemblies incorporated in chemical sensors and biosensors.
